# Bile-derived exosome noncoding RNAs as potential diagnostic and prognostic biomarkers for cholangiocarcinoma

**DOI:** 10.3389/fonc.2022.985089

**Published:** 2022-08-24

**Authors:** Yan Pan, Shijie Shao, Hang Sun, Huafeng Zhu, Haixing Fang

**Affiliations:** ^1^ Department of Integrative Oncology, The First People’s Hospital of Fuyang, Fuyang First Hospital Affiliated to Zhejiang Chinese Medical University, Hangzhou, China; ^2^ Department of Oncological Surgery, The First People’s Hospital of Fuyang, Fuyang First Hospital Affiliated to Zhejiang Chinese Medical University, Hangzhou, China; ^3^ The Second Clinical College, Zhejiang Chinese Medical University, Hangzhou, China

**Keywords:** bile, cholangiocarcinoma, exosome, lncRNA, microRNA, biomarker

## Abstract

**Background:**

Cholangiocarcinoma (CCA) is one of the most aggressive malignancies, lacking novel diagnostic and prognostic biomarkers. Exosome noncoding RNAs (ncRNA) were previously proposed as a potential source of biomarkers in several cancers. This study aimed to interpret the value of specific bile-derived ncRNA as predictors for early diagnosis and prognosis of CCA.

**Methods:**

We recruited 100 patients who received endoscopic retrograde cholangiopancreatography at our hospital for bile duct obstruction due to CCA (n = 50) and biliary stone (n = 50). They were further divided into training set and validation set (3:2). A panel of CCA-specific ncRNAs including 5 miRNAs (PMID: 30165035) and 2 lncRNAs (PMID: 29050258) were detected in both serum and bile exosomes. The diagnostic accuracy was assessed using the area under the receiver operating characteristic curve. Logistic analysis was used to classify the potential predictors of CCA and further establish the diagnostic model. And the prognostic value of the ncRNAs was also assessed.

**Results:**

Exosomes were successfully collected from bile and serum. Exosomal miR-141-3p, miR-200a-3p, miR-200c-3p in serum and bile, as well as miR-200b-3p and ENST00000588480.1 in bile showed AUCs of >0.70 in the diagnosis of CCA. Bile exosomal miR-200c-3p displayed the best diagnostic value with the AUC of 0.87. The combination of serum CA19-9 into the model could increase the AUC to 0.906. Bile exosomal miR-200a-3p and miR-200c-3p were found to be independent predictors of CCA. Among exosomal ncRNAs in human bile and blood, 3 (serum and bile exosomal miR-200c-3p, bile exosomal miR-200a-3p) showed significant value in predicting cancer recurrence and 1 (serum exosomal miR-200c-3p) had great predictive ability of cancer death. High levels of serum exosomal miR-200c-3p showed unfavorable tumor-free survival and overall survival.

**Conclusion:**

The bile exosomal miR-200 family, particularly miR-200c-3p, was verified to be a potential biomarker for the early detection of CCA. The diagnostic ability of exosomal ncRNAs in human bile is better than that in blood. Moreover, high levels of bile exosomal miR-200a-3p, miR-200c-3p, and serum exosomal miR-200c-3p represented adverse clinical outcomes.

## Introduction

Cholangiocarcinoma (CCA) is the second most common primary hepatic malignancy arising from the epithelium of the bile ducts and can involve any part of the biliary tract (1). It is far more prevalent in Asia, where the incidence is as high as 113 per 100,000 in some regions (2). CCA is an incurable and lethal cancer due to its silent clinical characteristics, difficulty in early diagnosis, with a 5-year survival of < 10% (3). Only approximately 30% patients diagnosed with CCA have the opportunity for curative resection because of the failure of early diagnosis (4). Therefore, new diagnostic and therapeutic approaches for CCA are urgently needed.

Exosomes, named small size vesicles (40–100nm), are secreted by multiple types of cells into body fluids, containing many kinds of ncRNAs and play an important role in human physiological and pathological development (5). In recent years, tumor-derived exosomes are emerging as a new type of cancer biomarker, and increasing evidences demonstrated that it could be a significant regulatory mechanism in the development, progression, and metastasis of cancer (6). Therefore, the signaling molecules (e.g., microRNA, lncRNA, circRNA, mRNA, and protein), carried by exosomes have displayed promising value in cancer early diagnosis, treatment assessment, and prognosis prediction. One of the miRNA families, the miR-200 family, was mainly characterized as a tumor suppressor, and composed of five highly conserved miRNAs, including miR-141, miR-200a/200b/200c and miR-429 (7). A published study showed that the expression of miR-200c was associated with unfavorable survival outcomes in some cancers, specifically in lung cancer and breast cancer (7). Our previous research confirmed that serum exosomal miR-200 family, particularly miR-200c-3p, could be efficient biomarkers for CCA, and the levels of serum exosomal miR-200a/c-3p could represent the rate of CCA progression (8).

CCA is originated from cholangiocytes, therefore, bile is a rich source of biomarkers for CCA potentially. In a previous study, bile was proved to contain exosome vesicles (EVs), to convey specific tumor- derived miRNAs (9). It has been also confirmed that EVs in bile were able to affect cholangiocyte proliferation (10) and high concentrations of EVs in bile were strongly associated with malignant common bile duct (CBD) stenosis (11). Another study demonstrated that exosomal lncRNAs of ENST00000588480.1 and ENST00000517758.1 could be potential diagnostic and therapeutic targets for CCA (12).

Therefore, in this study, we attempted to obtain exosomal 7 ncRNAs including 5 miRNAs and 2 lncRNAs from bile and serum and further identified novel biomarkers for early detection and prognosis of CCA.

## Materials and methods

### Clinical data and sample preparation

A total of 50 pathologically confirmed CCA patients undergoing endoscopic retrograde cholangiopancreatography (ERCP) in the First People’s Hospital of Fuyang from Jan 2017 to Dec 2019 were enrolled. Meanwhile, another 50 age- and gender-matched patients diagnosed as biliary stone disease on ERCP manipulation were randomly recruited as the control group.

The enrolled patients of each group were further randomly divided into a training set and a validation set according to 3:2 ratio. Patients’ characteristics are shown in **Table 1**.

**Table 1:** Patient characteristics.

	Training set		Validation set	
	CCA (n = 30)	Control (n = 30)	CCA (n = 20)	Control (n = 20)
Age (yr)	61.7 ± 8.8	61.0 ± 9.6	58.6 ± 9.9	59.8 ± 10.3
Male	16	16	11	11
Female	14	14	9	9
HBV	7	9	4	6
Overweight	2	3	2	1
Diabetes	2	4	1	2
Hypertension	5	4	3	3
Acute cholangitis	1	11	0	4
CA19-9 positive	19	5	13	2
Maximal tumor size (cm)	2.7 ± 2.2	NA	2.2 ± 1.9	NA
Tumor stage				
I-II	20	NA	12	NA
III-IV	10	NA	8	NA
Differentiation				
High/moderate	18	NA	13	NA
Low	12	NA	7	NA
Vascular invasion				
Macro-	2	NA	2	NA
Micro-	13	NA	8	NA
Location				
Intrahepatic	18	NA	11	NA
Extrahepatic	12	NA	9	NA
Treatment				
Hepatectomy + chemo	11	NA	7	NA
Whipple + chemo	13	NA	9	NA
Chemo	6	NA	4	NA
Resection margin				
R0	18	NA	12	NA
R1	6	NA	4	NA

Overweight: BMI>25; Acute cholangitis was defined as fever, abdominal pain, and jaundice;

Tumor stage was defined according to the TNM classification for CCA in the American Joint Committee on Cancer Staging manual (8th edition);

CA19-9, carbohydrate antigen 19-9; CA19-9 positive: > 37 U/ml; chemo, chemotherapy;

CCA, cholangiocarcinoma; HBV, hepatitis B virus; NA, not available.

In this study, serum and bile samples were obtained from every patient before treatment. 5ml of venous blood samples were collected before ERCP, centrifuged at 3000g for 10 minutes. Serum was obtained and transferred to RNase-free tubes, and stored at -80°C. Approximately 5ml of bile was aspirated after successful ERCP cannulation of the biliary tree before contrast injection, centrifuged at 4000g for 15 minutes. Supernatant was taken and stored in -80°C. After disease assessment, CCA patients with stage I-III underwent hepatectomy or whipple procedure according to the location of the lesion followed by adjuvant systemic chemotherapy based on capecitabine. Patients with unresectable and metastatic cancer were subjected to gemcitabine with cisplatin. If cancer progressed during or after systemic therapy, then FOLFOX was the preferred option. The project protocol was supported and approved by the Ethics Committee of the First People’s Hospital of Fuyang. All patients enrolled in the present research provided written informed consent.

### Exosome isolation and identification

Bile and serum exosomes from CCA patients and benign subjects were isolated using the miRCURY Exosome Kit (Qiagen, Valencia, CA, USA) according to the manufacturer’s protocol. 5ml of bile or serum was thawed and diluted with an equal volume of phosphate buffered saline (PBS), and centrifuged at 500g for 10 minutes at 4°C to pellet cells and debris. Then the supernatant was taken and centrifuged at 16500g for 20 minutes; filtered with a 0.22um syringe filter; transferred to a new tube, and placed on ice. Then we added precipitation buffer, mixed by pipetting until the samples were completely mixed. The samples were incubated at room temperature for 1 hour, and centrifuged 10000g for 30 minutes at 4°C. Ultimately, the obtained precipitate was exosomes. A drop of exosomes was pipetted onto a grid for 5 min at room temperature, then negatively stained with 3% (wt/vol) phosphotungstic acid (pH 6.8) for 5 min and analyzed after air drying under an electric incandescent lamp.

### RNA extraction and detection

Total RNAs were extracted from exosomes using Exosomal RNA Isolation kit (Norgen, Canada). Then, RNA was reverse transcribed using SuperScript II Reverse Transcriptase kit (Invitrogen, Carlsband, CA, USA). MicroRNA detection was performed using SYBRPrimeScript microRNA RTPCR Kit (TaKaRa, Dalian, China) according to the standard protocol. 5 previously identified CCA-associated miR-200 family members (8) (miR-141-3p, miR-200a-3p, miR-200b-3p, miR-200c-3p, and miR-205-5p) and 2 lncRNAs (ENST00000588480.1 and ENST00000517758.1) (12) were detected. Quantitative real-time polymerase chain reaction was performed using an SYBR kit in an Applied Biosystems 7900 Sequence Detection System (Life Technologies, Carlsbad, CA, USA). The PCR primers were listed in **Table 2**.

**Table 2:** The PCR primers.

ID	Primers
hsa-miR-200a-3p	F: 5’-GCCGGGTAACACTGTCTGGTA-3’ F: 5’-GTCGTATCCAGTGCAGGGTCCGAGGTATTCGCACTGGATACGACACATCGT-3’
hsa-miR-200b-3p	F: 5’-GCCGGGTAATACTGCCTGGTAA-3’ R: 5’-GTCGTATCCAGTGCAGGGTCCGAGGTATTCGCACTGGATACGACTCATCA-3’
hsa-miR-200c-3p	F: 5’-GCCGGGTAATACTGCCGGGTAAT-3’ R: 5’-GTCGTATCCAGTGCAGGGTCCGAGGTATTCGCACTGGATACGACATCCATC-3’
hsa-miR-141-3p	F: 5’-GCCGGGTAACACTGTCTGGTAA-3’ R: 5’-GTCGTATCCAGTGCAGGGTCCGAGGTATTCGCACTGGATACGACATCCCT-3’
hsa-miR-205-5p	F: 5’-GCCGGGTCCTTCATTCCACCGG-3’ R: 5’-GTCGTATCCAGTGCAGGGTCCGAGGTATTCGCACTGGATACGACCAGACT-3
ENST00000588480.1	GGCTTTCCGCAGTGAACTCGGGGGCACTATCCTCGGTACCCAGATGCCCACCTGTGTGCC ACTCCCCTCCCAAACCAGCCCCTGAGCCCGAGCTCTGTTACCTGCAGCGAACTCCTCGAT GGTCATGAGCGGGAAGCGAATGAGGCCCAGGGCCTTGCCCAGAACCTTCCGCCTGTTCTC TGGCGTCACCTGCAGCTGCTGCCGCTGACACTCGGCCTCGGACCAGCGGACAACGGCATT GAACAGCCGCACCTCACGGATGCCCAGTGTGTCGCGCTCCAGGACAGCCACCAGCGTGTC TGTGGGGTGGAGGAAGGGGCTGCGTGAACACGACACCCACATGCCCACCCTGCAGGAGGC AGTGAGACTAACGTGAGGACCAGCAGTTAAAGCCGGGCTGGCAACTATGGCCCGTGGCCC AAATCTGGCCCTGTGAGTGTGTTTATAAATAAAGCTTTATCAACACAA F: 5’-GAACCTTCCGCCTGTTCTCT-3’ R: 5’-CGTTAGTCTCACTGCCTCCTG-3’
ENST00000517758.1	TTTCAGGTAGTTGGGAGCCAGGGAAGGAGTGACACTATCCCTAGATGTAGTGATATGGTT TTCCTGGGAGGGCTTGACTTAATCAGGTGAGCCCTTTAAATGGATTGGGACCCTCATGGA GGTCAGATGTGTTCTCCTGCTGACCTTGAAAATGTAATCTGCAATGTGAACGTGTTGATG GAGGGGGCCAAATGCCAAGGACCTGAGGGGGCACCGTGGGATCTGAGAGTAGTCCGCAGC TAATAACTAGCAAGAAAATGAAACCCAACACTTAGCCACAAGAAACTGAATTATGCTAAC AACTATTTGAGCTTTGAAGAGGATCCTGAGCACCAGAAAGTAATGTCGCATAGCAACCAC GTTGATTGCATCCTTGCAAGACCTCGTGATGACCACTCAATTAATTCATGCCCAGATTCA TGCCCATTGAAACTGTGATAATACATGTATGTTGTTTTACATTA F: 5’-TTCAGGTAGTTGGGAGCCAG-3’ R: 5’-GCTGCGGACTACTCTCAGAT-3’

### Statistical analysis

All data were presented as mean ± standard deviation (SD) and compared using Student t-test. Categorical variables were presented as values (percentages) and compared by Pearson’s χ2 test. Areas under the receiver operating characteristic curve (AUCs) were applied to evaluate the predictive value of selected ncRNAs for CCA. Cutoff value was chosen by considering the diagnostic sensitivity and specificity. Logistic analysis was used to identify the valid biomarkers of CCA and establish the diagnostic model. SPSS version 17.0 (SPSS Inc., Chicago, IL) was used to complete the statistical analyses. A *P* value <0.05 was considered statistically significant.

## Results

### Bile exosomal ncRNAs could be used to distinguish CCA from benign biliary diseases

**Figure 1:**
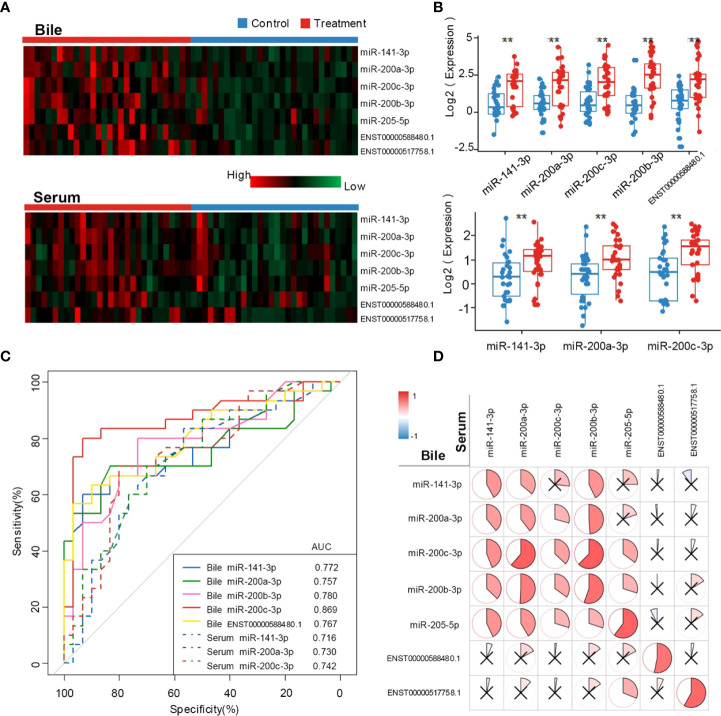
Exosomal ncRNAs in human bile and serum. **(A)** Heatmap showing the expression of exosomal ncRNAs in human bile and serum; **(B)** Significantly differential expressed exosomal ncRNAs between CCA group and control group; **(C)** The AUCs of exosomal ncRNAs in the diagnosis of CCA; **(D)** The correlation between serum and bile exosomal ncRNAs. **: vs. control group.

Patient characteristics in the training set are shown in **Table 1**. Compared with the control group, CCA group showed a significantly higher CA19-9 positive rate (*P* < 0.001) and lower incidence of acute cholangitis (*P* = 0.002). Exosomal ncRNAs were successfully obtained from the bile and compared between CCA group and control group (**Figure 1A**). As shown in the training set (**Figures 1A**, **B**), the relative expression levels of bile exosomal miR-141-3p, miR-200a-3p, miR-200b-3p, miR-200c-3p and ENST00000588480.1 were significantly increased in patients with CCA compared with the controls (all *P <*0.05). No statistical significance was found for the expression of miR-205-5p and ENST00000517758.1 between the two groups. The diagnostic value of the upregulated five ncRNAs was estimated by ROC curve analyses through synthesizing the results of 60 participants (30 CCA and 30 controls). The area under the curve (AUC) of all selected ncRNAs was >0.75 in the diagnosis of CCA (**Figure 1C**; **Table 3**). Exosomal miR-200c-3p displayed the best diagnostic value with the AUC of 0.87 (**Figure 1C**; **Table 3**).

**Table 3:** Comparison of AUCs in the diagnosis of CCA.

Bile exosomal ncRNAs	Sensitivity (%)	Specificity (%)	AUC
miR-141-3p	70.0	63.3	0.772^*^
miR-200a-3p	70.0	83.3	0.757^*^
miR-200b-3p	80.0	73.3	0.780^*^
miR-200c-3p	83.3	86.7	0.869
miR-205-5p	63.3	63.3	0.676
ENST00000588480.1	70.0	66.7	0.767^*^
ENST00000517758.1	66.7	60.0	0.690
**Serum exosomal ncRNAs**			
miR-141-3p	73.3	63.3	0.716^*^
miR-200a-3p	70.0	70.0	0.730^*^
miR-200b-3p	56.7	50.0	0.552
miR-200c-3p	76.7	63.3	0.742^*^
miR-205-5p	60.0	60.0	0.636
ENST00000588480.1	73.3	50.0	0.643
ENST00000517758.1	73.3	60.0	0.664

^*^: vs. Bile exosomal miR-200c-3p.

### The diagnostic value of exosomal ncRNAs in human bile is better than those in blood

To compare the diagnostic capability of exosomal ncRNAs in human bile and blood, the same seven exosomal ncRNAs were also detected in serum from CCA group and control group (**Figure 1A**). As displayed in **Figure 1B**, only three ncRNAs (miR-141-3p, miR-200a-3p, and miR-200c-3p) were significantly differently expressed between the two groups. In the diagnosis of CCA, all three serum ncRNAs presented AUCs of > 0.70 (**Figure 1C**; **Table 3**). MiR-200c-3p showed the highest AUC of 0.742 (**Figure 1C**; **Table 3**). After comparison, it was evident that the AUCs of the detected ncRNAs in the bile samples were higher than those of serum samples. Bile exosomal miR-200c-3p showed the best diagnostic ability (**Figure 1C**; **Table 3**). In addition, the same ncRNA from serum and bile were significantly related. Different members of the miR-200 family including miR-200a-3p, miR-200b-3p and miR-200c-3p were also significantly consistent in bile and serum (**Figure 1D**). Therefore, the bile ncRNAs had a more efficient diagnostic value than that of serum.

### Integrated diagnostic model

We next assessed the diagnostic value of conventional biomarkers (CA19-9, CA12-5, and CEA) in both serum and bile. Serum CA19-9 and bile CEA presented AUCs of > 0.70. All serum and bile compounds with AUCs of > 0.70 were considered potential diagnostic biomarkers of CCA and then entered a stepwise logistic analysis (**Table 4**). Ultimately, bile exosomal miR-200a-3p and miR-200c-3p were found to be independent predictors of CCA. A diagnostic model (model 1) that integrated with bile exosomal miR-200a-3p and miR-200c-3p showed a sensitivity of 83.3%, specificity of 86.7% and AUC of 0.897 in the training set.

**Table 4:** Potential biomarkers of CCA.

	Univariate		Multivariate	
	*P*	OR (95%CI)	*P*	OR (95%CI)
Serum CA19-9 > 37 U/ml	<.001	8.636 (2.566, 29.07)		
Bile CEA > 25 ng/ml	0.044	3.763 (1.038, 13.65)		
Serum exosomal miR-141-3p	0.011	4.030 (1.372, 11.84)		
Serum exosomal miR-200a-3p	0.003	5.444 (1.804, 16.43)		
Serum exosomal miR-200c-3p	0.003	5.675 (1.841, 17.49)		
Bile exosomal miR-141-3p	0.005	4.750 (1.584, 14.25)		
Bile exosomal miR-200a-3p	<.001	11.67 (3.384, 40.22)	0.027	5.581 (1.213, 25.68)
Bile exosomal miR-200b-3p	<.001	11.00 (3.292, 36.75)		
Bile exosomal miR-200c-3p	<.001	32.50 (7.818, 135.1)	<.001	20.69 (4.631, 92.43)
Bile exosomal ENST00000588480.1	0.006	4.667 (1.571, 13.87)		
Bile exosomal ENST00000517758.1	0.041	3.000 (1.046, 8.603)		

CA19-9, carbohydrate antigen 19-9; CEA, carcinoembryonic antigen; CCA, cholangiocarcinoma; OR, odds ratio, CI, confidential interval.

The combination of serum CA19-9 into the model (model 2) could increase the AUC to 0.906, but did not change the sensitivity and specificity. These two diagnostic models were further verified in the validation group, which enrolled 20 CCA patients and 20 controls (**Table 1**). Both models showed a sensitivity of 85.0% and specificity of 85.0%. The AUC of model 1 and model 2 were 0.880 and 0.893, respectively.

### The clinical prognostic value of exosomal ncRNAs in human bile and blood

**Figure 2:**
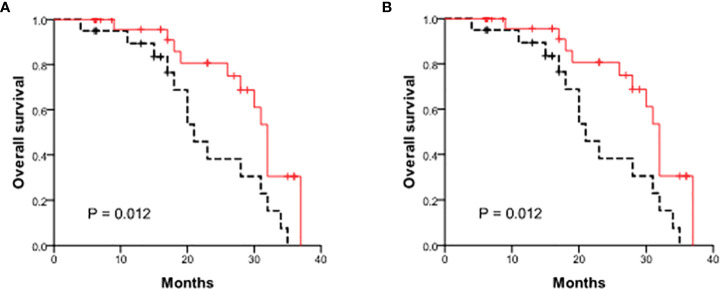
Comparison of patient survival between patients with high and low levels of serum exosomal miR-200c-3p. **(A)** Tumor-free survival curves; **(B)** The overall survival curves.

We further investigated the prognostic value of exosomal ncRNAs in human bile and blood in all 50 CCA patients. The cumulative tumor-free survival rates were 74.0% and 43.7% at 1- and 2-year. The patient survival rates were 93.1% and 63.6% at 1- and 2-year. Cox hazard regression was performed to identify the risk factors for cancer recurrence and mortality. Among the 7 exosomal ncRNAs both in human bile and blood, 3 (serum and bile exosomal miR-200c-3p, bile exosomal miR-200a-3p) showed significant value in predicting cancer recurrence and 1 (serum exosomal miR-200c-3p) had great predictive ability of cancer death (**Table 5**). High levels of serum exosomal miR-200c-3p (above average) showed significantly decreased tumor-free survival and overall survival (**Figure 2**).

**Table 5:** Influencing factors of patient survival.

	Tumor-free survival		Overall survival	
	*P*	RR (95%CI)	*P*	RR (95%CI)
Tumor differentiation	0.036	4.689 (1.109, 1.787)		
Tumor stage	0.031	2.371 (1.085, 5.185)	0.017	3.396 (1.247, 9.253)
Serum exosomal miR-200c-3p	0.026	1.155 (1.018, 1.311)	0.047	1.383 (1.005, 1.904)
Bile exosomal miR-200a-3p	0.047	2.292 (1.010, 5.198)		
Bile exosomal miR-200c-3p	0.039	2.688 (1.052, 6.897)		

CCA, cholangiocarcinoma; RR, risk ratio, CI, confidential interval.

## Discussion

CCA is widely acknowledged as an aggressive type of liver neoplasms hard to discover (13). To find solutions to the problem, a large number of studies have been published in the past decade, regarding the potential role of exosomes in CCA as diagnostic biomarkers (14–16). Exosomes in body fluids are relatively stable (17, 18) and widely involved in cancer cell proliferation, invasion, angiogenesis, and metastasis *via* endocrine and paracrine mechanisms (19). Among all types of exosomal ncRNAs, miRNAs are considered fundamental regulators of gene expression and play an important role in tumorigenesis, metastasis, and resistance to various therapies (19). In addition, exosomal miRNAs are more stable than circulating blood miRNAs (20). The miR-200 family is one of the best investigated functional miRNAs and is mainly recognized as tumor suppressors (7). In our previous study, we proved serum exosomal miR-200c-3p, a member of miR-200 family, was an ideal biomarker for early diagnosis of CCA and might be associated with CCA progression (8).

Theoretically, exosomes are more concentrated in bile than that in serum because they are secreted by CCA cells before releasing into the blood. In a previous study, bile was proved to contain exosome vesicles (EVs), to convey specific tumor-derived miRNAs (21). It has been also confirmed that EVs in bile were able to affect cholangiocyte proliferation (10) and high concentrations of EVs in bile were strongly associated with malignant CBD stenosis (11). Thereafter, we hypothesized that exosomes in bile, higher in concentration compared with serum, could be predictive biomarkers and are more effective in the early diagnosis of CCA.

To find the answer, the current research was conducted in several steps. Firstly, we successfully obtained the exosomes from bile and then detected targeted ncRNAs (5 exosomal miR-200 family members and 2 lncRNAs) to evaluate the diagnostic value for CCA. This study confirmed that the expression level of 4 exosomal miR-200 family members (miR-141-3p, miR-200a-3p, miR-200b-3p, and miR-200c-3p) in bile was significantly higher in the CCA group than in the control group with UACs of >0.75, indicating the potential diagnostic value of those exosomal miRNAs. Bile exosomal miR-200c-3p displayed the best diagnostic value with the AUC of 0.869. As for lncRNAs, we found ENST00000588480.1 was significantly upregulated in exosomes isolated from bile samples of CCA than biliary stone patients, which was partially consistent with Ge’s results (12). Thus, ENST00000588480.1 could be a marker for CCA diagnosis with the AUC of 0.767. Secondly, these results confirmed the hypothesis that the diagnostic ability of exosomal ncRNAs in human bile is better than those in serum with a maximum AUC of 0.869 for CCA. Another published study showed similar results that exosomes in bile samples could discriminate between patients with malignant *vs* nonmalignant CBD stenosis with 100% accuracy (11). Thirdly, we combined the diagnostic value of serum CA19-9 with bile exosomal miR-200a-3p and miR-200c-3p, which could increase the AUC to 0.906. In comparison, the sensitivity and specificity of CA19-9 are not ideal, especially in the early stage of the disease. Recent meta-analysis of CA19-9 as a marker for the diagnosis of CCA showed that its sensitivity and specificity were less than 80% (22). In the diagnosis of intrahepatic CCA, the sensitivity and specificity of CA19-9 were only 62% and 63% (23). In addition, CA19-9 is susceptible to biliary tract infection, benign obstructive jaundice, and other factors when used as a diagnostic marker for early screening of CCA (24). In short, these results confirmed our hypothesis that the exosomal miR-200 family in bile displayed stronger ability in the early diagnosis of CCA than those in serum, especially miR-200c-3p. Combining miRNAs with CA19-9 could enhance the reliability of CCA diagnosis.

Moreover, we found that bile exosomal miR-200a-3p, miR-200c-3p, and serum exosomal miR-200c-3p were not only diagnostic biomarkers but also signs representing unfavorable patients’ survival, closely associated with tumor stage and tumor differentiation. Plenty of published articles showed that higher expression of miR-200 family was related to worse survival outcomes for cancer patients (7, 25). It was reported in a meta-analysis that miR-200 expression was negatively associated with survival prognosis in all cancers combined, especially in lung and breast cancer (7). In breast cancer, up-regulated miR-200 represents more prone to liver metastasis (26, 27). The miR-200c/141 cluster upregulated SerpinB2 in the MDA-MB-231 breast cancer cell line, inducing cell metastasis, which partially explains the mechanism (28). In ovarian cancer (29), a previous study indicated that miR-200 family members might affect the β-tubulin III protein and negatively regulate EphA2 expression. Oppositely, miR-200 family members are proved to be tumor suppressors, which are significantly involved in the inhibition of epithelial-to-mesenchymal transition (EMT) (30).

Although bile miRNAs were more effective than serum miRNAs in the early diagnosis of CCA, serum miRNAs were better predictors of overall survival. We hypothesized that the elevation of serum miRNAs could better reflect the overall situation of the patient, just like the tumor stage. While the bile miRNAs only reflected the local situation of the tumor. Therefore, the prediction effects of bile miRNAs and tumor differention were more effective than that of serum miRNAs in predicting tumor-free survival.

In conclusion, our research suggested that the bile exosomal miR-200 family, especially miR-200c-3p, could be an efficient biomarker for the early detection of CCA. The diagnostic capability of exosomal ncRNAs in human bile is better than that in blood. Additionally, higher levels of bile exosomal miR-200a-3p, miR-200c-3p, and serum exosomal miR-200c-3p indicated worse clinical outcomes. However, well-designed, large-scale prospective studies are needed to verify these findings due to the limited cases in this study.

## Data availability statement

The authors acknowledge that the data presented in this study must be deposited and made publicly available in an acceptable repository, prior to publication. Frontiers cannot accept a manuscript that does not adhere to our open data policies.

## Ethics statement

This study was reviewed and approved by The Ethics Committee of the First People’s Hospital of Fuyang. The patients/participants provided their written informed consent to participate in this study.

## Author contributions

HF substantial contributions to the design of the work and agrees to be accountable for all aspects of the work in ensuring that questions related to the accuracy or integrity of any part of the work are appropriately investigated and resolved. YP conducts the work, analyzes of data for the work and revises the article critically. SS draft the article critically. HS and HZ provides approval for publication of the content. All authors contributed to the article and approved the submitted version.

## Funding

This research was supported by Zhejiang Provincial Natural Science Foundation of China under Grant No. LGF21H160016.

## Conflict of interest

The authors declare that the research was conducted in the absence of any commercial or financial relationships that could be construed as a potential conflict of interest.

## Publisher’s note

All claims expressed in this article are solely those of the authors and do not necessarily represent those of their affiliated organizations, or those of the publisher, the editors and the reviewers. Any product that may be evaluated in this article, or claim that may be made by its manufacturer, is not guaranteed or endorsed by the publisher.

## References

[1] Tyson GL El-Serag HB. Risk factors for cholangiocarcinoma. Hepatology (2011) 54(1):173–84. doi: 10.1002/hep.24351 PMC3125451 21488076

[2] Khan AS Dageforde LA. Cholangiocarcinoma. Surg Clin North Am (2019) 99(2):315–35. doi: 10.1016/j.suc.2018.12.004 30846037

[3] Bergquist A von Seth E. Epidemiology of cholangiocarcinoma. Best Pract Res Clin Gastroenterol (2015) 29(2):221–32. doi: 10.1016/j.bpg.2015.02.003 25966423

[4] Marsh Rde W Alonzo M Bajaj S Baker M Elton E Farrell TA. Comprehensive review of the diagnosis and treatment of biliary tract cancer 2012. part I: diagnosis-clinical staging and pathology. J Surg Oncol (2012) 106(3):332–8. doi: 10.1002/jso.23028 22488652

[5] Liang B Zhong L He Q Wang S Pan Z Wang T. Diagnostic accuracy of serum CA19-9 in patients with cholangiocarcinoma: A systematic review and meta-analysis. Med Sci Monit (2015) 21:3555–63. doi: 10.12659/MSM.895040 PMC4655615 26576628

[6] Ruivo CF Adem B Silva M Melo SA. The biology of cancer exosomes: Insights and new perspectives. Cancer Res (2017) 77(23):6480–8. doi: 10.1158/0008-5472.CAN-17-0994 29162616

[7] Huang GL Sun J Lu Y Liu Y Cao H Zhang H. MiR-200 family and cancer: From a meta-analysis view. Mol Aspects Med (2019) 70:57–71. doi: 10.1016/j.mam.2019.09.005 31558294

[8] Shen L Chen G Xia Q Shao S Fang H. Exosomal miR-200 family as serum biomarkers for early detection and prognostic prediction of cholangiocarcinoma. Int J Clin Exp Pathol (2019) 12(10):3870–6. PMC6949744 31933776

[9] Li L Masica D Ishida M Tomuleasa C Umegaki S Kalloo AN. Human bile contains microRNA-laden extracellular vesicles that can be used for cholangiocarcinoma diagnosis. Hepatology (2014) 60(3):896–907. doi: 10.1002/hep.27050 24497320 PMC4121391

[10] Masyuk AI Huang BQ Ward CJ Gradilone SA Banales JM Masyuk TV. Biliary exosomes influence cholangiocyte regulatory mechanisms and proliferation through interaction with primary cilia. Am J Physiol Gastrointest Liver Physiol (2010) 299(4):G990–9. doi: 10.1152/ajpgi.00093.2010 PMC2957333 20634433

[11] Severino V Dumonceau JM Delhaye M Moll S Annessi-Ramseyer I Robin X. Extracellular vesicles in bile as markers of malignant biliary stenoses. Gastroenterology (2017) 153(2):495–504.e498. doi: 10.1053/j.gastro.2017.04.043 28479376

[12] Ge X Wang Y Nie J Li Q Tang L Deng X. The diagnostic/prognostic potential and molecular functions of long non-coding RNAs in the exosomes derived from the bile of human cholangiocarcinoma. Oncotarget (2017) 8(41):69995–70005. doi: 10.18632/oncotarget.19547 29050258 PMC5642533

[13] Siegel RL Miller KD Fuchs HE Jemal A. Cancer statistics, 2021. CA: Cancer J Clin (2021) 71(1):7–33. doi: 10.3322/caac.21654 33433946

[14] Olaizola P Lee-Law PY Arbelaiz A Lapitz A Perugorria MJ Bujanda L. MicroRNAs and extracellular vesicles in cholangiopathies. Biochim Biophys Acta Mol Basis Dis (2018) 1864(4 Pt B):1293–307. doi: 10.1016/j.bbadis.2017.06.026 28711597

[15] Xue XY Liu YX Wang C Gu XJ Xue ZQ Zang XL. Identification of exosomal miRNAs as diagnostic biomarkers for cholangiocarcinoma and gallbladder carcinoma. Signal Transduct Targeted Ther (2020) 5(1):77. doi: 10.1038/s41392-020-0162-6 PMC7289871 32527999

[16] Arbelaiz A Azkargorta M Krawczyk M Santos-Laso A Lapitz A Perugorria MJ. Serum extracellular vesicles contain protein biomarkers for primary sclerosing cholangitis and cholangiocarcinoma. Hepatol (Baltimore Md) (2017) 66(4):1125–43. doi: 10.1002/hep.29291 28555885

[17] Malik ZA Kott KS Poe AJ Kuo T Chen L Ferrara KW. Cardiac myocyte exosomes: stability, HSP60, and proteomics. Am J Physiol Heart Circ Physiol (2013) 304(7):H954–65. doi: 10.1152/ajpheart.00835.2012 PMC3625894 23376832

[18] Gezer U Ozgur E Cetinkaya M Isin M Dalay N. Long non-coding RNAs with low expression levels in cells are enriched in secreted exosomes. Cell Biol Int (2014) 38(9):1076–9. doi: 10.1002/cbin.10301 24798520

[19] Dilsiz N. Role of exosomes and exosomal microRNAs in cancer. Future Sci OA (2020) 6(4):FSO465. doi: 10.2144/fsoa-2019-0116 32257377 PMC7117563

[20] Kitdumrongthum S Metheetrairut C Charoensawan V Ounjai P Janpipatkul K Panvongsa W. Dysregulated microRNA expression profiles in cholangiocarcinoma cell-derived exosomes. Life Sci (2018) 210:65–75. doi: 10.1016/j.lfs.2018.08.058 30165035

[21] Han JY Ahn KS Kim YH Kim TS Baek WK Suh SI. Circulating microRNAs as biomarkers in bile-derived exosomes of cholangiocarcinoma. Ann Surg Treat Res (2021) 101(3):140–50. doi: 10.4174/astr.2021.101.3.140 PMC8424434 34549037

[22] Macias RIR Kornek M Rodrigues PM Paiva NA Castro RE Urban S. Diagnostic and prognostic biomarkers in cholangiocarcinoma. Liver Int (2019) 39 Suppl 1:108–22. doi: 10.1111/liv.14090 30843325

[23] Macias RIR Banales JM Sangro B Muntane J Avila MA Lozano E. The search for novel diagnostic and prognostic biomarkers in cholangiocarcinoma. Biochim Biophys Acta Mol Basis Dis (2018) 1864(4 Pt B):1468–77. doi: 10.1016/j.bbadis.2017.08.002 28782657

[24] Fang T Wang H Wang Y Lin X Cui Y Wang Z. Clinical significance of CA125, and CA19-9 levels in predicting the resectability of cholangiocarcinoma. Dis Markers (2019) 2019:6016931. doi: 10.1155/2019/6016931 30863466 PMC6378785

[25] Liu W Zhang K Wei P Hu Y Peng Y Fang X. Correlation between miR-200 family overexpression and cancer prognosis. Dis Markers (2018) 2018:6071826. doi: 10.1155/2018/6071826 30069274 PMC6057334

[26] Antolin S Calvo L Blanco-Calvo M Santiago MP Lorenzo-Patino MJ Haz-Conde M. Circulating miR-200c and miR-141 and outcomes in patients with breast cancer. BMC Cancer (2015) 15:297. doi: 10.1186/s12885-015-1238-5 25885099 PMC4405843

[27] Debeb BG Lacerda L Anfossi S Diagaradjane P Chu K Bambhroliya A. miR-141-Mediated regulation of brain metastasis from breast cancer. J Natl Cancer Inst (2016) 108(8):djw026. doi: 10.1093/jnci/djw026 PMC5017951 27075851

[28] Jin T Suk Kim H Ki Choi S Hye Hwang E Woo J Suk Ryu H. microRNA-200c/141 upregulates SerpinB2 to promote breast cancer cell metastasis and reduce patient survival. Oncotarget (2017) 8(20):32769–82. doi: 10.18632/oncotarget.15680 PMC5464826 28427146

[29] Sun Q Zou X Zhang T Shen J Yin Y Xiang J. The role of miR-200a in vasculogenic mimicry and its clinical significance in ovarian cancer. Gynecol Oncol (2014) 132(3):730–8. doi: 10.1016/j.ygyno.2014.01.047 24503464

[30] Feng X Wang Z Fillmore R Xi Y. MiR-200, a new star miRNA in human cancer. Cancer Lett (2014) 344(2):166–73. doi: 10.1016/j.canlet.2013.11.004 PMC3946634 24262661

